# The Milk Metabolome of Non-secretor and Lewis Negative Mothers

**DOI:** 10.3389/fnut.2020.576966

**Published:** 2021-02-02

**Authors:** Aidong Wang, Petya Koleva, Elloise du Toit, Donna T. Geddes, Daniel Munblit, Susan L. Prescott, Merete Eggesbø, Christine C. Johnson, Ganesa Wegienka, Naoki Shimojo, Dianne Campbell, Anita L. Kozyrskyj, Carolyn M. Slupsky

**Affiliations:** ^1^Department of Food Science and Technology, University of California, Davis, Davis, CA, United States; ^2^InVivo Planetary Health of the Worldwide Universities Network (WUN), West New York, NJ, United States; ^3^Department of Pediatrics, University of Alberta, Edmonton, AB, Canada; ^4^Department of Pathology, University of Cape Town, Cape Town, South Africa; ^5^School of Molecular Sciences, University of Western Australia, Perth, WA, Australia; ^6^Paediatrics and Paediatric Infectious Diseases, Institute of Child's Health, Sechenov First Moscow State Medical University (Sechenov University), Moscow, Russia; ^7^Section of Inflammation, Repair and Development, National Heart and Lung Institute, Imperial College London, London, United Kingdom; ^8^The ORIGINS Project, Telethon Kids Institute, Perth Childrens Hospital, University of Western Australia, Crawley, WA, Australia; ^9^Department of Environmental Exposure and Epidemiology, Norwegian Institute of Public Health, Oslo, Norway; ^10^Department of Public Health Sciences, Henry Ford Health System, Detroit, MI, United States; ^11^Center for Preventive Medical Sciences, Chiba University, Chiba, Japan; ^12^Department of Allergy and Immunology, Children's Hospital at Westmead, University of Sydney, Sydney, NSW, Australia; ^13^Department of Nutrition, University of California, Davis, Davis, CA, United States

**Keywords:** human milk, metabolome, Lewis negative, oligosaccharide, energy metabolism, South Africa, non-secretor

## Abstract

**Introduction:** The functional role of milk for the developing neonate is an area of great interest, and a significant amount of research has been done. However, a lot of work remains to fully understand the complexities of milk, and the variations imposed through genetics. It has previously been shown that both secretor (Se) and Lewis blood type (Le) status impacts the human milk oligosaccharide (HMO) content of human milk. While some studies have compared the non-HMO milk metabolome of Se+ and Se− women, none have reported on the non-HMO milk metabolome of Se− and Le– mothers.

**Method and Results:** To determine the differences in the non-HMO milk metabolome between Se–Le– mothers and other HMO phenotypes (Se+Le+, Se+Le–, and Se–Le+), 10 milk samples from 10 lactating mothers were analyzed using nuclear magnetic resonance (NMR) spectroscopy. Se or Le HMO phenotypes were assigned based on the presence and absence of 6 HMOs generated by the Se and Le genes. After classification, 58 milk metabolites were compared among the HMO phenotypes. Principal component analysis (PCA) identified clear separation between Se–Le– milk and the other milks. Fold change analysis demonstrated that the Se–Le– milk had major differences in free fatty acids, free amino acids, and metabolites related to energy metabolism.

**Conclusion:** The results of this brief research report suggest that the milk metabolome of mothers with the Se–Le– phenotype differs in its non-HMO metabolite composition from mothers with other HMO phenotypes.

## Introduction

Human milk is the gold standard for infant nutrition as it provides essential nutrients for infant growth, as well as bioactive components such as human milk oligosaccharides (HMOs). While the variation of HMOs among different maternal HMO phenotypes has been widely studied (1–4), the impact of the maternal HMO phenotypes on other low-molecular-weight milk metabolites remains unclear. Metabolites other than oligosaccharides are thought to play important roles in infant health. For example, milk glutamate has been shown to impact appetite and growth (5), biogenic amines have been reported to provide protection against infectious disease (6), taurine has been recognized to contribute to neonatal brain development (7), and creatine appears to be essential for normal neural development (8). An understanding of how these metabolites change with HMO phenotype may be important to further understanding of the function of these metabolites in milk.

Maternal HMO phenotypes are determined by the activity of two genes: the secretor (Se) gene *fut2*, coding for α-1,2-fucosyltransferase 2 (FUT2), and the Lewis (Le) gene *fut3*, coding for α-1,3/1,4-fucosyltransferase (FUT3). FUT2 and FUT3 are responsible for the fucosylation of milk oligosaccharides. There are five monosaccharides upon which all HMOs are built: D-glucose, D-galactose, N-acetylglucosamine (GlcNAc), L-fucose and sialic acid (Neu5Ac) (9). At the core of the HMO structure is lactose, which can be sialylated to form α2-3 (e.g., 3′sialyllactose, 3'SL) or α2-6 (e.g., 6′sialyllactose, 6'SL) linkages to sialic acid, or fucosylated to form α1-2 (e.g., 2′FL), or α1-3 (e.g., 3FL) linkages to fucose. To form more complex HMOs, lactose can be elongated through a β1-3 linkage to lacto-N-biose (type I) or a β1-6 linkage to N-acetyllactosamine (type II). Lactose or the formed polylactosamine backbone can then be sialylated and/or fucosylated to create an additional 200 different oligosaccharide structures (10). FUT2 synthesizes 2'FL or lacto-N-fucopentose I (LNFP I) by attaching a fucose to lactose or lacto-N-tetraose (LNT), respectively. FUT3 synthesizes lacto-N-difucohexaose I (LDFH I) and lactodifucotetraose (LDFT) from LNFP I and 2'FL, respectively, by attaching an additional fucose. FUT3 can also directly transfer fucose to LNT, lactose, and lacto-N-neotetraose (LNnT) to form lacto-N-neotetraose II (LNFP II), 3FL, and lacto-N-neotetraose III (LNFP III), respectively (4). Additionally, the α-1,3-fucosyltransferases encoded by *fut4, 5, 6, 7*, and/or *9*, which are Se− and Le− independent, also play roles in attaching fucose to lactose, and thus 3FL and LNFP III can sometimes be observed in milk from Lewis negative (Le−) women (11, 12). It has been speculated that FUT1 α-1,2-fucosyltransferase 1 also participates in HMO fucosylation, as α-1-2-fucosylated HMOs have been observed in milk from Se− women (13). In human milk from Se+/Le+ women, 35–50% of the HMOs are fucosylated, 12–14% are sialylated, and 42–55% are non-fucosylated neutral (14).

While the Se and Le genes are important to generate a variety of HMOs in both free and conjugated forms, many individuals have polymorphisms in one or both of these genes making them non-functional. In European and American populations, the Le− frequency is between 4 and 6%, and 20% of the population are Se–, making Se–Le– extremely rare. In contrast, in certain African populations, over 30% of the population are Le− and ~38% are Se− (15–17), which makes the probability of having Se–Le– mothers higher. The importance of functional Se and Le genes in infant development is an area of active research. One study showed that maternal secretor status appeared to be important for preventing diarrhea, as although the gut microbiota measured through 16S rRNA sequencing did not differ between infants of Se+ and Se− mothers, the prevalence of diarrhea was higher among infants of Se− mothers (18). Moreover, when these infants were provided iron supplements, infants of Se− mothers were more likely to experience a decrease in the abundance of *Bifidobacterium* and an increase in pathogens compared to infants of Se+ mothers (18). However, supplementation with galactooligosaccharides appeared to ameliorate the impact of iron supplementation (18).

Studies comparing the non-HMO milk metabolome from mothers who were phenotypically Se+ to Se− demonstrated no differences between groups (19, 20). We have previously reported on the milk metabolome at day 90 (21) and over the first month of lactation (22) in Se+Le+ and Se–Le+ women. We observed no significant difference in non-HMO metabolites between the two groups. To date, no studies have compared the non-HMO metabolome of milk from phenotypically Se–Le– mothers to any other phenotype. We hypothesized that the non-HMO milk metabolome from Se–Le– women would be similar to the other phenotypes 1 month after delivery. This brief research report provides preliminary data on the comparison of the milk metabolome between women with the Se–Le– phenotype and other phenotypes.

## Methods

### Milk Sample Preparation

In this pilot study, to maximize the homogeneity of subjects (23), human milk samples were collected 1 month postpartum from 10 randomly-selected healthy women (age 29.8 ± 4.8, pre-pregnancy BMI 25.0 ± 2.9) in Cape Town, South Africa, who gave birth to term infants (50% male) through vaginal delivery, and practiced exclusive breastfeeding prior to sample collection. The exclusion criteria included antibiotic or probiotic treatment during the last trimester of pregnancy, and the breastfeeding period. Ethical approval for this study was provided by the University of Cape Town's Human Research Ethical Committee (HREC REF: 306/2014). Mature milk samples from mothers were collected after obtaining their consent. Women were asked to wash their hands, their nipple, and surrounding breast area with soap, then soak the breast area with chlorhexidine to reduce contamination by skin microbes, followed by washing with sterile water. A small volume of milk was collected manually or with an electric breast pump into a sterile collection bottle after discarding the first few drops. Time since last feed was not recorded. After collection, samples were transported on ice and stored at −20°C until further processing. This study is a subset of a larger study on the relationship of milk short chain fatty acids and atopy (24).

Milk samples were prepared as previously described (25). Briefly, samples were thawed on ice, mixed, then 500 μL of each sample was filtered through Amicon Ultra 0.5 mL 3-kDa cutoff spin filters (Millipore Sigma, Burlington, MA, USA) at 10,000 × g for 15 min at 4°C to remove lipids and protein, as the study was interested in low-molecular-weight polar metabolites. Three hundred and fifty microliter of filtrate was mixed with 70 μL of deuterium oxide and 60 μL of standard buffer solution [consisting of 585 mM NaHPO4 (pH 7.0), 11.667 mM disodium-2,2-dimethyl-2-silapentane-5-sulfonate (DSS, internal standard), and 0.47% NaN_3_ in H_2_O] in a 1.5 mL Eppendorf tube (25). Four hundred and sixty microliter of the mixture was transferred to a nuclear magnetic resonance (NMR) tube for subsequent NMR spectral analysis.

### NMR Data Acquisition and Processing

^1^H NMR spectra were acquired at 25°C using the first transient of the Varian tnnoesy pulse sequence on a Varian 500 MHz Inova spectrometer equipped with a 5 mm HCN cold probe. Water suppression pulses were calibrated to achieve a bandwidth of 80 G. Spectra were collected with 128 transients and 8 steady-state scans using a 4 s acquisition time (48,000 complex points) and a 1 s recycle delay. Before spectral analysis, all free induction decays were zero-filled to 64,000 data points and line broadened to 0.5 Hz. The methyl singlet produced by DSS internal standard was used for chemical shift referencing (set to 0 ppm) and for quantification. Spectra were manually processed and 64 polar milk metabolites (including the 6 HMOs used for phenotype determination) were identified and quantified using Chenomx NMRSuite version 8.1 (Chenomx Inc., Edmonton, AB, Canada).

### HMO Phenotype Determination

The HMO phenotype was determined based on the presence or absence of six specific milk oligosaccharides (2'FL, 3FL, LNFP I, LNFP II, LNFP III, and LDFT) in the NMR spectra that were identified and quantified from an NMR spectral library created through the analytical preparation of commercially available HMOs as previously described (21). In this study, the limit of detection was set to 20 μM for these compounds based on the ability to clearly observe spectral peaks of these HMOs over noise in the spectra generated from the Varian 500 MHz Inova spectrometer. Detection of both 2'FL and LNFP I in milk resulted in phenotype assignment as Se+, otherwise Se–. When LNFP II, 3FL, LDFT, and LNFP III were visible in the NMR spectra, the phenotype was assigned as Le+, otherwise Le–.

### Statistical Analysis

Statistical computing and graphical generation were performed using the R (version 3.5.2) programing environment. Prior to principal component analysis (PCA), generalized log transformation [defined as log_2_(1+y) where y is the metabolite concentration] was applied to all metabolomics data. PCA was computed using the *prcomp* function in the *stats* package of R without scaling the transformed data, and the first two components were plotted.

Metabolomics data without log transformation was used to perform log2_Fold calculation according the following equation. Briefly, the mean concentration of each metabolite was first calculated for the Se–Le–, Se–Le+, and Se+ phenotypes (the Se+Le+ and Se+Le– samples were combined since there was only one Se+Le– sample). The mean concentration of each metabolite in the Se–Le– (or Se–Le+) groups was divided by the mean concentration of the same metabolite in the Se+ group to calculate the ratio between Se–Le– (or Se–Le+) and Se+ phenotypes. To ensure metabolites were expressed in the same range, log2 transformation was applied. To decrease the chance of false discovery using FDR-corrected *p*-values (since most metabolites were significantly different using this method), we considered a log2 fold change cut off of ±1.5 as an indication of significance.

## Results

**Figure 1:**
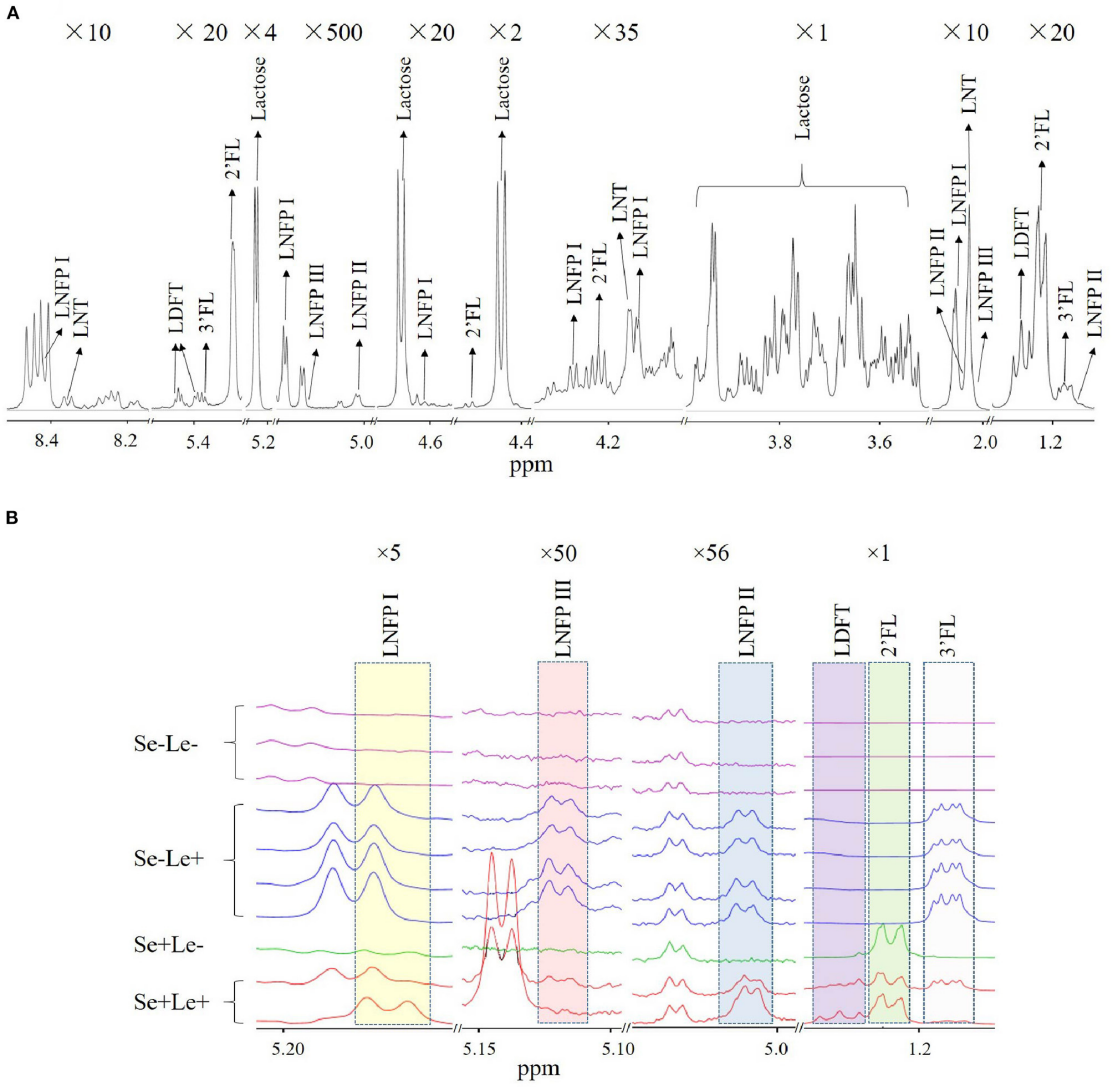
Identification of HMOs in human milk NMR spectra. **(A)** Multiple peaks of each HMO are shown in 10 different chemical shift regions at various vertical scales to illustrate characteristic peaks associated with identified HMOs. Magnification is indicated at the top of each segment. **(B)** Comparison of NMR spectra of milk between individuals with putative differences in Se and Le status.

In total, 10 milk samples were collected from South African women 1 month after term delivery, of which 60% (*n* = 6) were Mixed Race, 20% (*n* = 2) were Black, and 20% (*n* = 2) were Caucasian. None of the women had atopic disease. An NMR spectrum annotated with HMO peaks is shown in Figure 1A. Multiple peaks of each HMO could be identified, with some overlapping with other metabolites in milk. The HMO phenotypes of the subjects was estimated by assessing the presence or absence of specific HMOs in the milk samples (Table 1), with examples of the NMR spectrum corresponding to each of the HMO phenotypes shown in Figure 1B. Samples where both 2'FL and LNFP I could be measured were assigned as Se+, while samples where these two HMOs could not be detected were designated Se–. No sample was detected with only one of the two HMOs. Se+ samples with the presence of LNFP II, 3FL, LDFT, and LNFP III were assigned as Se+Le+, otherwise they were assigned as Se+Le–. Se− samples with detectable levels of LNFP II, 3FL, and LNFP III were classified as Se–Le+, and for those without these three HMOs as Se–Le–. Out of 10 samples analyzed, three samples were designated Se–Le–, as none of the six targeted HMOs was detected in any of these samples. Additionally, the area under the peak for the three FUT 3-catalyzed HMOs (LNFP II, LNFP III and 3FL) were higher in milk from Se–Le+ mothers compared to milk from Se+Le+ mothers (Figure 1B).

**Table 1:** Proposed synthetic pathways of the principal fucosyloligosaccharides used to identify Secretor (Se) and Lewis (Le) phenotypes based on their presence/absence in the 10 milk samples.

	**Starting structure**	**LNT**	**Lactose**	**LNT**	**LNnT**	**Lactose**	**2'FL**	
**Subject**	**HMO phenotype**	**Se+**		**Le+**				**Proposed HMO phenotype**
	**HMO**	**LNFP I**	**2'FL**	**LNFP II**	**LNFP III**	**3FL**	**LDFT**	
1		bld	bld	bld	bld	bld	bld	Se–Le–
2		√	√	√	√	√	√	Se+Le+
3		bld	bld	√	√	√	bld	Se–Le+
4		bld	bld	√	√	√	bld	Se–Le+
5		bld	bld	bld	bld	bld	bld	Se–Le–
6		bld	bld	√	√	√	bld	Se–Le+
7		bld	bld	√	√	√	bld	Se–Le+
8		bld	bld	bld	bld	bld	bld	Se–Le–
9		√	√	bld	bld	bld	bld	Se+Le–
10		√	√	√	√	√	√	Se+Le+

*LNFP I, lacto-N-fucopentose I; LNFP II, lacto-N-fucopentose II; LNFP III, lacto-N-fucopentose III; LNT, lacto-N-tetraose; LNnT, lacto-N-neotetraose; LDFT, lactodifucotetraose; 2'FL, 2'fucosyllactose; 3FL, 3-fucosyllactose; Se, secretor; Le, Lewis; √, Detected; bld, Below limit of detection. HMO concentrations below 20 μM were considered below the detection limit*.

**Figure 2:**
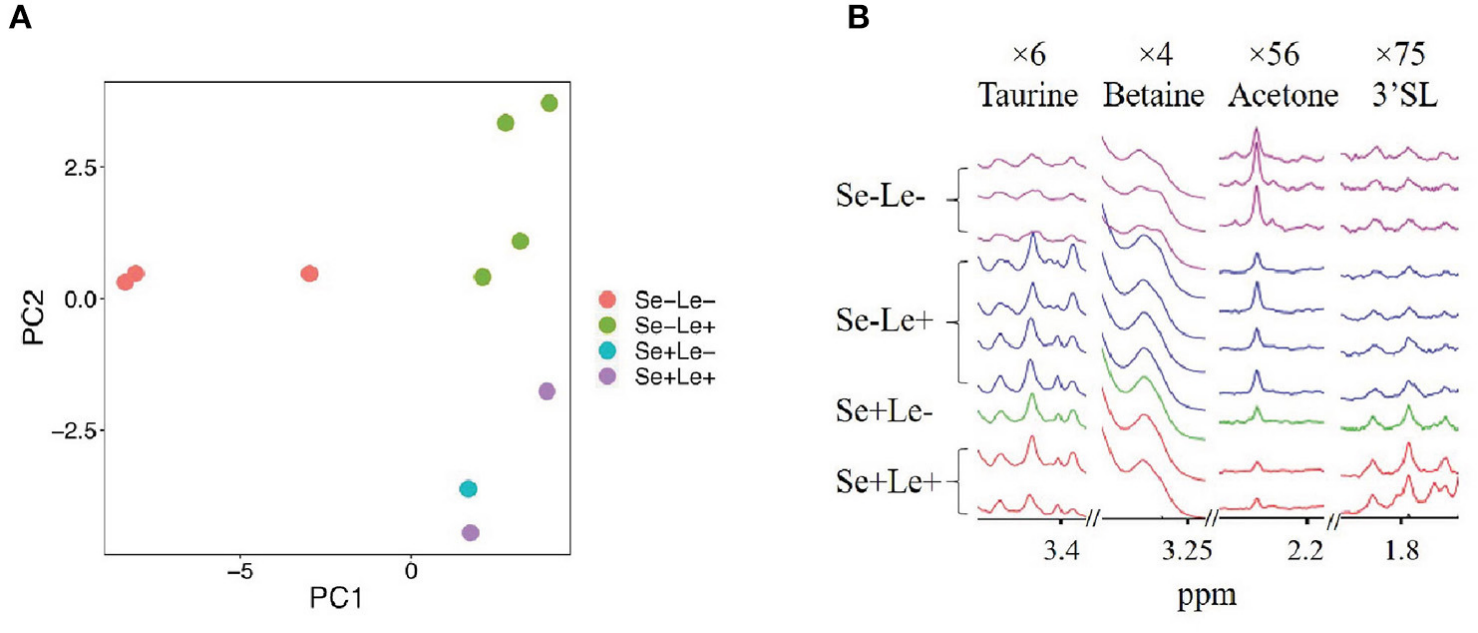
Comparison of non-HMO milk metabolites between milk from women with different Se and Le status. **(A)** Principal component analysis of metabolites not used for HMO phenotype assignment. **(B)** Comparison of some non-HMO milk metabolites between Se-Le- and other phenotypes. Magnification is indicated at the top of each segment.

To evaluate whether the milk metabolome was different among the HMO phenotypes, 58 quantified polar metabolites (excluding the HMOs resulting from FUT2 and FUT3) were examined and compared. Figure 2A shows a principal component analysis (PCA) of milk metabolites of women from the identified HMO phenotypes. Separation along PC1, which explained 48.7% of the variance, revealed a difference between the Se–Le– group and all other groups. Along PC2, which explained 15.0% of the variance, separation based on Se status was observed. As there was only one sample identified as Se+Le–, and it did not separate from the Se+Le+ samples in the PCA plot (Figure 2A), it was combined with the Se+Le+ samples (Se+ samples) in further analyses.

**Figure 3:**
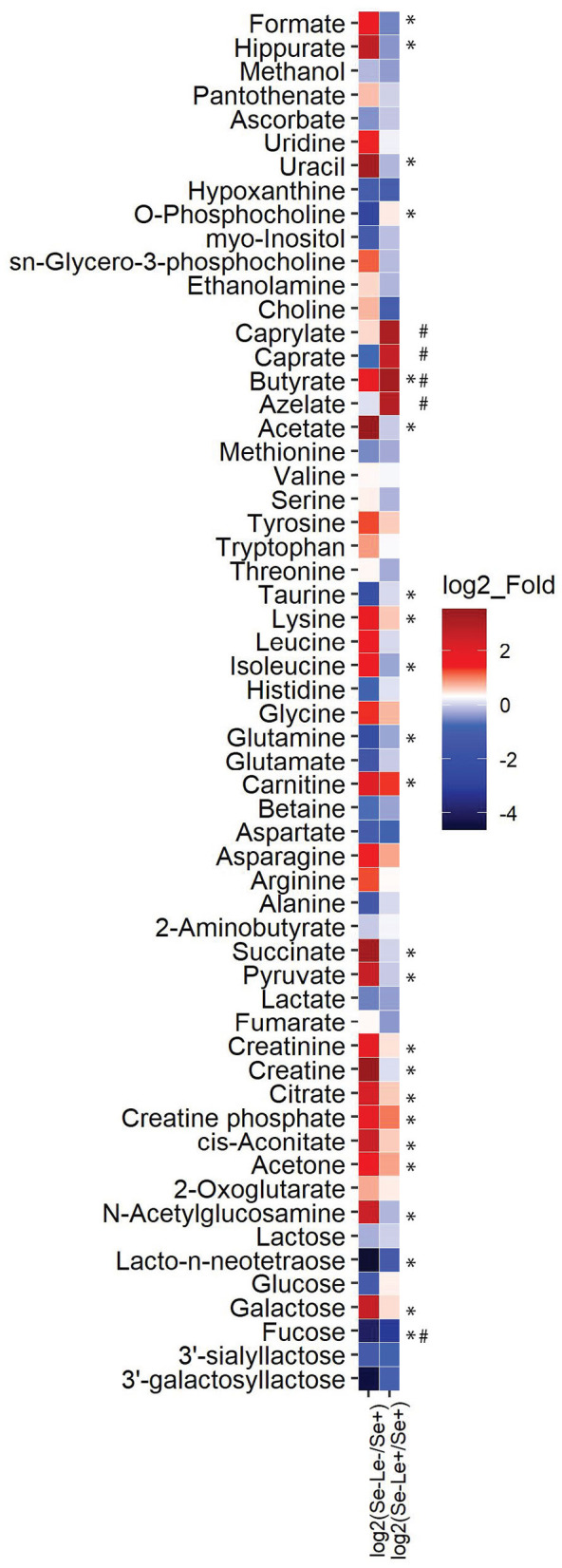
Fold difference of metabolite concentrations in milk from Se–Le– and Se–Le+ mothers relative to Se+ mothers. The mean concentration of each metabolite was calculated for all groups and the means of the Se–Le– and Se–Le+ groups were divided by the mean of the metabolite concentration from the Se+ groups to determine the ratio relative to the Se+ groups. The ratio values were then log2 transformed. Log2_fold values over 1.5 or below −1.5 are indicated in the figure. * log2_fold over 1.5 or below −1.5 when comparing Se–Le– to Se+ samples. ^#^ log2_fold over 1.5 or below −1.5 when comparing Se–Le+ to Se+ samples.

In order to further compare milk metabolites among groups, the fold/ratio of metabolite concentrations in milk from Se–Le– and Se–Le+ mothers relative to milk from Se+ mothers were calculated (Figure 3). In terms of the oligosaccharides and their metabolites, 3'galactosyllactose, 3'SL, fucose, and LNnT were between 2- and 10-fold lower in milk samples from Se–Le– and Se–Le+ compared to Se+ mothers. Galactose was 6 and 1 times higher in milk samples from Se–Le– and Se–Le+ mothers, respectively, compared to samples from Se+ mothers. For metabolites associated with energy metabolism, samples from Se–Le– milk were approximately 4 times higher in creatine phosphate, 12 times higher in creatine, 4 times higher in creatinine, 5 times higher in citrate, 6 times higher in pyruvate, and 10 times higher in succinate compared to Se+ milk, while these metabolites were similar in concentration between milk from Se–Le+ and Se+ mothers.

Milk from Se–Le– mothers also differed with respect to free amino acid concentrations compared to milk from Se+ and Se–Le+ mothers. Arginine, asparagine, glycine, leucine, isoleucine, lysine, and tyrosine were 2–4-fold higher in milk samples from Se–Le– compared to Se+. Interestingly, the fold difference of these amino acids in milk between Se–Le+ and Se+ samples was <2-fold. Carnitine was higher in milk from both the Se–Le– (~4 fold) and Se–Le+ (~2 fold) groups compared to Se+. Alanine, glutamate, glutamine, taurine, and betaine were all between 2- and 5-fold lower in the Se–Le– group compared to the Se+ group, while they were similar in concentration between the Se–Le+ and Se+ groups. Aspartate was also 2-fold lower in milk from both Se–Le– and Se–Le+ samples compared to Se+.

Free fatty acids and associated metabolites such as acetate, choline, and sn-glycero-3-phosphocholine were 12-, 2-, and 2-fold higher, respectively, in the Se–Le– group compared to samples from Se+ mothers. Azelate, butyrate, caprate, and caprylate were also 8-, 10-, 6-, and 9-fold higher respectively in the Se–Le+ group compared to Se+ samples. Additionally, butyrate was 3- and 10-fold higher in Se–Le– and Se–Le+ groups compared to Se+. O-phosphocholine was lower in the Se–Le– group (10-fold) compared to Se+. Representative peaks of taurine, betaine, acetone and 3'SL are shown in Figure 2B. Metabolite concentrations for each subject are shown in Supplementary Table 1.

## Discussion

Significant research has been undertaken to understand the impact of maternal secretor status and Lewis blood type on the milk glycome (12) and subsequent influence on infant health including their gut microbiota (26), susceptibility to rotavirus (15), allergy to bovine milk (27), and weight during the first 6 months (28). But no studies have focused on the metabolome of milk from the Se–Le– population due to its low prevalence.

In the current study, we found that all of the 6 fucosylated HMOs used to determine HMO phenotypes were below the detection limit of the instrument in Se–Le– samples. 3FL and LNFP III were previously reported to be present in milk from Le− women, which could potentially be due to the activity of FUT4, 5, 6, 7, and 9 enzymes (1). In the current study, neither of these HMOs was detected in Le− women, which may be due to the difference in detection methods. Mass Spectrometry can measure down to the picomolar level, whereas for spectra obtained from the Varian 500 MHz spectrometer used in this study, the limit of detection of these metabolites was 20 μM. It remains to be determined if oligosaccharides present in milk below 20 μM would have a significant impact on infant health.

Other HMOs and related metabolites such as 3′galactosyllactose, 3′SL, fucose, galactose, and GlcNAc also trended different within the HMO phenotypes, indicating other factors influencing the glycome of human milk (Figure 3). 3′SL (Figure 2B), lower in both Se–Le– and Se–Le+ groups compared to Se+ in this study, was reported to be similar in concentration in milk from Se+ and Se− women (14, 21) or even ~20–56% higher in milk from Se− compared to Se+ women (18, 29). 6′-sialyllactose, which is not reported in the current study, was demonstrated to be significantly higher in milk from non-secretor women (14). Further studies are needed to investigate if this is due to a preference of α-2, 6-sialylation / α-2, 3-sialylation or simply a difference amongst populations.

Pyruvate, citrate, cis-aconitate, and succinate, which are metabolites involved in the tricarboxylic acid (TCA) cycle, were higher in Se–Le– milk. Increased TCA cycling could indicate greater energy provision, and previous studies have speculated that a higher level of TCA intermediates in bovine milk compared to human milk may be to enhance growth (30, 31).

HMO biosynthesis is suggested to be an extension of lactose biosynthesis which occurs in the Golgi of the mammary gland epithelial cells (32). Therefore, inactivity of both α-1,2- and α-1,3/1,4- fucosylltransferases in Se–Le– women might profoundly impact mammary gland metabolism, and thus impact milk composition. Alanine, taurine, glutamine and glutamate are the most abundant free amino acids in human milk (21, 33), and these were all lower in the milk from the Se–Le– group compared to milk from the Se+ group. Higher free glutamate in bovine milk infant formula has been reported to decrease its intake (5). It could be that a lower level of glutamate in milk from Se–Le– women could increase milk intake by the infant to compensate for the low and less diverse HMO content. Branched chain amino acids (leucine and isoleucine) and lysine were higher in Se–Le– compared to Se+ milk. A similar pattern of free amino acids in human milk was seen in a previous study comparing high and low growth rate groups of premature infants (34), where a higher content of insulinotrophic amino acids and tyrosine was associated with faster infant growth.

Choline in the Se–Le– group was almost double the level in Se+ samples, while phosphocholine was one-tenth the level. A previous study showed a negative correlation between choline and phosphocholine in human milk (35), and a similar correlation was observed in this study. The origin of choline in milk is not completely understood. One study reported that breastmilk choline is related to maternal choline intake and genetic polymorphisms (36), while another study showed no difference in milk choline content based on maternal diet (37). Indeed, the betaine level in Se–Le– milk was 2-fold lower than that in Se+ samples, suggesting a possible lower conversion of choline to betaine. It could be that the difference in milk choline (and other metabolites) in the Se–Le– group compared to Se+ group could result in differences in milk lipid synthesis (38). Indeed, choline is an essential precursor of phosphatidylcholine and sphingomyelin, which are essential components of biological membranes and precursors for intracellular messengers such as ceramide and diacylglycerol (39). This would imply that the milk fat globule would be different in Se–Le– mothers since maternal phenotype will impact conjugated glycolipids in addition to HMOs (40). Differences in the milk fat would need to be assessed in a separate study.

Here, in this brief research report, we showed differences in the non-HMO milk metabolome between phenotypically Se–Le– mothers and Se–Le+, and Se+ mothers. These differences included metabolites related to energy metabolism, amino acids, and fatty acids. The current study is limited by the small sample size and the rarity of Se–Le– HMO phenotype. Factors such as the completeness of milk expression, time since last feed, time of the day during sample collection, and information on mother's diet were not collected; however, the impact of these factors on milk composition is negligible compared to the impact of genetics. Nonetheless, this study shows that the Se and Le status of the mother has an important role to play in the composition of non-oligosaccharide milk metabolites. Further research involving larger sample sizes should be done to confirm the findings, investigate the impact on milk lipid and proteins, and investigate potential biological consequences of Se–Le– milk on infant gut microbial succession and metabolism. This will help further unravel the link between human milk and infant health.

## Data Availability Statement

The raw data supporting the conclusions of this article are available at: https://www.ebi.ac.uk/metabolights/MTBLS1899.

## Ethics Statement

The studies involving human participants were reviewed and approved by University of Cape Town's Human Research Ethical Committee. The patients/participants provided their written informed consent to participate in this study.

## Author Contributions

ET, DG, DM, SP, ME, CJ, GW, NS, DC, and AK designed the study. ET collected samples. PK prepared samples and collected NMR data. AW and CS analyzed and interpreted the data. AW drafted the manuscript. AK and CS provided funding. All authors edited and approved the manuscript.

## Conflict of Interest

The authors declare that the research was conducted in the absence of any commercial or financial relationships that could be construed as a potential conflict of interest.

## Abbreviations

HMOs: Human milk oligosaccharides
Se: secretor
Le: Lewis
FUT1: α-1,2-fucosyltransferase 1
FUT2: α-1,2-fucosyltransferase 2
FUT3: α-1,3/1,4-fucosyltransferase
GlcNAc: N-acetylglucosamine
Neu5Ac: sialic acid
LNFP I: lacto-N-fucopentose I
LNFP II: lacto-N-fucopentose II
LNFP III: lacto-N-fucopentose III
LNT: lacto-N-tetraose
LNnT: lacto-N-neotetraose
LDFH I: lacto-N-difucohexaose I
LDFH II: lacto-N-difucohesaose II
LDFT: lactodifucotetraose
2'FL: 2'fucosyllactose
3FL: 3-fucosyllactose
3'SL: 3'sialyllactose
6'SL: 6'sialyllactose
PCA: principal component analysis.
