# Supplementary material for: The Milk Metabolome of Non-secretor and Lewis Negative Mothers
Source: Front Nutr. 2021 Feb 2;7:576966. doi: 10.3389/fnut.2020.576966 (PMC7901958; doi:10.3389/fnut.2020.576966)
Supplement: Supplementary file 1 [file Data_Sheet_1.PDF]

Supplementary Table 1. Milk metabolite concentrations for each subject.

Sample ID	1	5	8	3	4	6	7	9	2	10
HMO phenotypes	Se-Le-			Se-Le+				Se+Le-	Se+Le+	
Metabolites (uM)										
2'fucosyllactose	bld	bld	bld	bld	bld	bld	bld	7259.1	5766.1	3392.7
2-Aminobutyrate	bld	bld	8.0	6.7	8.6	10.7	17.9	13.2	bld	11.1
2-Oxoglutarate	77.3	79.4	112.1	77.5	74.9	69.2	57.8	68.6	41.9	45.4
3-fucosyllactose	bld	bld	bld	7877.3	7510.0	4687.8	5293.1	bld	602.3	3377.6
3'galactosyllactose	bld	bld	25.5	187.4	168.9	158.7	46.6	27.4	786.9	bld
3'sialyllactose	117.0	124.5	126.1	175.0	183.7	114.5	126.5	242.0	288.1	286.3
Acetate	360.0	370.9	80.5	14.1	20.7	31.6	25.5	23.1	28.9	18.2
Acetone	22.6	24.0	16.6	12.9	11.3	15.7	10.1	9.2	5.8	6.2
Alanine	72.8	74.1	56.9	122.4	161.0	139.6	280.8	220.9	46.5	235.0
Arginine	90.2	92.8	30.5	53.5	36.7	28.9	32.4	48.3	29.9	bld
Ascorbate	91.6	91.6	62.8	94.4	159.4	81.7	88.3	140.1	122.9	67.6
Asparagine	29.5	29.5	31.1	bld	bld	38.0	26.5	11.9	bld	15.2
Aspartate	23.2	23.2	47.2	bld	42.5	39.9	44.1	101.7	29.1	67.2
Azelate	bld	12.1	24.6	28.6	108.5	172.7	161.9	bld	bld	25.6
Betaine	65.0	54.5	60.3	79.9	73.2	73.7	90.8	101.2	103.6	88.4
Butyrate	52.4	39.5	92.8	32.8	63.9	339.8	233.1	bld	bld	38.7
Caprate	bld	bld	16.8	69.3	74.1	97.8	224.9	9.6	24.2	22.0
Caprylate	27.5	27.5	38.8	97.6	86.6	250.3	331.6	bld	20.6	36.7
Carnitine	58.8	54.5	30.8	24.6	23.5	29.7	42.1	19.1	bld	bld
Choline	550.2	568.5	315.8	179.6	173.6	118.1	84.2	221.7	415.5	230.0
Citrate	11444.8	11615.2	11628.0	3788.6	3529.9	3754.1	4038.3	2074.1	3994.9	1350.1
Creatine	555.9	678.8	824.7	98.6	86.4	34.3	33.2	46.4	77.4	52.7
Creatine phosphate	bld	bld	179.4	29.5	32.2	39.3	40.8	11.4	15.7	24.3
Creatinine	220.8	230.7	81.6	78.6	75.8	53.3	59.7	45.7	49.4	48.8
Ethanolamine	188.5	231.4	73.1	109.2	127.4	74.1	89.2	122.9	136.8	78.2

(continued)

Table 1 continued

Sample ID	1	5	8	3	4	6	7	9	2	10
HMO phenotypes		Se-Le-			Se-Le+			Se+Le-	Se+Le+	
Metabolites (uM)										
Formate	145.8	144.2	38.1	21.4	27.4	27.4	22.8	26.0	69.3	bld
Fucose	18.2	27.9	29.4	73.8	69.7	14.0	21.7	437.7	229.1	531.2
Fumarate	bld	bld	22.4	bld	5.2	bld	7.1	8.5	5.2	6.1
Galactose	592.4	482.8	204.6	160.3	52.0	106.2	80.0	11.6	170.7	27.3
Glucose	493.4	275.3	709.5	951.3	1233.8	1494.1	2289.9	1447.0	386.0	1526.5
Glutamate	305.0	351.0	473.9	902.8	1022.0	996.1	1177.8	1736.1	199.6	1193.0
Glutamine	60.5	87.7	24.7	110.6	167.5	204.0	436.6	430.1	27.4	389.2
Glycine	590.5	590.5	174.9	112.5	266.6	403.4	383.2	180.3	228.0	123.1
Hippurate	86.0	88.5	121.5	16.1	bld	bld	11.9	bld	11.2	25.5
Histidine	bld	7.5	bld	11.0	bld	20.6	12.1	23.5	bld	bld
Hypoxanthine	bld	bld	6.2	bld	4.2	5.8	4.2	6.7	16.6	2.9
Isoleucine	24.2	26.5	8.8	9.0	7.2	bld	bld	11.2	bld	6.6
Lacto-N-fucopentaose II	bld	bld	bld	363.5	306.2	388.7	563.9	bld	324.1	175.4
Lactate	180.6	191.5	130.5	235.3	198.9	128.6	207.7	186.5	356.6	189.0
Lacto-N-fucopentaose I	bld	bld	bld	bld	bld	bld	bld	503.8	1810.8	64.2
Lacto-N-fucopentaose III	bld	bld	bld	243.5	199.2	179.3	172.7	bld	35.0	106.4
Lacto-N-neotetraose	28.7	bld	30.4	335.3	352.8	288.4	87.7	424.4	1368.3	46.7
Lactodifucotetraose	bld	bld	bld	bld	bld	bld	bld	bld	445.9	823.9
Lactose	177325.2	173715.2	191447.1	201532.0	204280.0	214368.3	222308.8	215690.1	189847.9	225892.4
Leucine	59.3	63.7	17.1	20.7	19.3	16.2	14.4	21.5	9.3	19.7
Lysine	57.6	58.2	12.3	21.5	23.5	17.8	15.5	16.3	13.2	bld
Methanol	86.1	84.0	97.0	82.5	71.8	75.3	80.6	84.3	86.8	125.5
Methionine	17.9	18.7	bld	15.0	13.9	17.4	17.9	22.0	14.2	21.1
N-Acetylglucosamine	784.9	899.2	779.0	83.5	89.9	166.9	168.2	45.0	338.6	46.8
O-Phosphocholine	37.8	41.4	137.0	518.0	575.0	797.8	834.0	349.8	543.3	614.0
Pantothenate	22.5	16.4	21.7	13.2	14.7	10.5	12.6	13.6	bld	18.6

(continued)

Table 1 continued

Sample ID	1	5	8	3	4	6	7	9	2	10
HMO phenotypes	Se-Le-			Se-Le+				Se+Le-	Se+Le+	
Metabolites (uM)										
Pyruvate	93.9	92.7	51.9	15.3	11.7	10.1	15.0	8.3	21.4	10.3
Serine	903.2	1143.4	841.8	524.7	720.2	344.3	940.1	487.0	993.8	684.3
Succinate	160.7	165.5	30.5	12.1	11.6	13.7	12.0	9.6	15.8	11.0
Taurine	123.0	124.8	89.4	620.7	632.2	403.3	339.5	535.4	382.1	513.5
Threonine	28.9	17.9	8.5	8.4	18.2	10.9	10.5	11.6	13.9	17.1
Tryptophan	27.4	21.1	bld	9.0	10.7	12.3	13.4	10.6	8.2	9.4
Tyrosine	39.6	41.0	bld	24.1	24.6	12.4	13.4	14.7	bld	14.0
Uracil	69.9	70.6	22.9	bld	bld	15.7	bld	bld	10.3	bld
Uridine	6.5	bld	63.9	8.2	6.7	23.9	4.4	15.0	7.5	bld
Valine	35.4	38.0	19.9	32.1	31.2	27.7	23.3	33.3	7.5	31.3
cis-Aconitate	60.8	66.5	19.1	bld	12.7	17.2	12.4	10.6	bld	9.2
myo-Inositol	391.8	400.5	231.1	692.6	566.9	786.8	749.6	334.8	1366.7	554.0
sn-Glycero-3-phosphocholine	1257.2	1274.1	1476.9	463.4	461.8	621.3	621.0	803.8	475.7	503.1

Bld: below detection limit
